# Docosane-Organosilica Microcapsules for Structural Composites with Thermal Energy Storage/Release Capability

**DOI:** 10.3390/ma12081286

**Published:** 2019-04-19

**Authors:** Giulia Fredi, Sandra Dirè, Emanuela Callone, Riccardo Ceccato, Francesco Mondadori, Alessandro Pegoretti

**Affiliations:** 1 Department of Industrial Engineering and INSTM research unit, University of Trento, Via Sommarive 9, 38123 Trento, Italy; emanuela.callone@unitn.it (E.C.); riccardo.ceccato@unitn.it (R.C.); francesco.mondadori93@gmail.com (F.M.); 2 “Klaus Müller” Magnetic Resonance Lab., Department of Industrial Engineering, University of Trento, via Sommarive 9, 38123 Trento, Italy

**Keywords:** microencapsulated phase change materials, thermal energy storage, sol–gel organosilica, nuclear magnetic resonance, multifunctional composites, thermal properties

## Abstract

Organic phase change materials (PCMs) represent an effective solution to manage intermittent energy sources as the solar thermal energy. This work aims at encapsulating docosane in organosilica shells and at dispersing the produced capsules in epoxy/carbon laminates to manufacture multifunctional structural composites for thermal energy storage (TES). Microcapsules of different sizes were prepared by hydrolysis-condensation of methyltriethoxysilane (MTES) in an oil-in-water emulsion. X-ray diffraction (XRD) highlighted the difference in the crystalline structure of pristine and microencapsulated docosane, and ^13^C solid-state nuclear magnetic resonance (NMR) evidenced the influence of microcapsules size on the shifts of the representative docosane signals, as a consequence of confinement effects, i.e., reduced chain mobility and interaction with the inner shell walls. A phase change enthalpy up to 143 J/g was determined via differential scanning calorimetry (DSC) on microcapsules, and tests at low scanning speed emphasized the differences in the crystallization behavior and allowed the calculation of the phase change activation energy of docosane, which increased upon encapsulation. Then, the possibility of embedding the microcapsules in an epoxy resin and in an epoxy/carbon laminate to produce a structural TES composite was investigated. The presence of microcapsules agglomerates and the poor capsule-epoxy adhesion, both evidenced by scanning electron microscopy (SEM), led to a decrease in the mechanical properties, as confirmed by three-point bending tests. Dynamic mechanical analysis (DMA) highlighted that the storage modulus decreased by 15% after docosane melting and that the glass transition temperature of the epoxy resin was not influenced by the PCM. The heat storage/release properties of the obtained laminates were proved through DSC and thermal camera imaging tests.

## 1. Introduction

Using phase change materials (PCMs) for thermal energy storage (TES) has become increasingly frequent in the last decades, as PCMs help to effectively manage intermittent energy sources, such as in the solar-thermal power plants or the solar-thermal systems for temperature regulation of buildings and water heating [1].

In these applications, PCMs have proven to be effective renewable energy materials, able to reduce the mismatch between energy availability and demand, thereby increasing the efficiency in energy utilization and reducing emissions [2,3,4]. Their superiority over the other TES systems, i.e., sensible heat and thermochemical heat materials, stems from their ability to store a high energy amount per unit mass in a narrow temperature interval. The most widely used PCMs, particularly in the low-medium temperature range (0–120 °C), are organic materials, such as paraffins, poly(ethylene glycol)s and fatty acids. Their main advantages are represented by the large availability, tunable working temperature, low density, cheapness, chemical inertness and non-corrosiveness [5,6,7]. However, two main drawbacks must be considered, namely the low thermal conductivity and the leakage above the melting temperature [8,9]. The latter issue can be addressed in several ways, such as the encapsulation and the shape stabilization with porous/layered materials or polymer matrices [10,11]. Among these, the most common is the encapsulation of the PCM in inert micro- or nano-shells, which not only avoid the loss of molten PCM due to leakage, but also enhance the heat transfer by increasing the specific surface area, protect the PCM from the external environment, increase the compatibility with the surrounding matrix, control the volume change during phase transition, and also improve the thermal conductivity and the thermal stability [12,13,14]. 

The capsule shell can be made of polymeric or inorganic material. Polymeric shells (acrylate polymers, polystyrene, melamine-formaldehyde resins, urea-formaldehyde resins, siloxane polymers [14,15,16,17,18]) are often the preferred choice, as they can be prepared with well consolidated and industrially available chemical techniques, such as coacervation or interfacial/emulsion/suspension polymerization [13,19]. The main advantage of polymeric shells is the low density, which allows a high core-to-shell weight ratio and therefore an enhanced total TES performance. On the other hand, organic shells are often highly flammable, can release toxic gases, and exhibit lower thermal and mechanical stability and lower thermal conductivity than their inorganic counterparts [20]. The most common inorganic shell material is silica [21], but many research studies have been carried out also on calcium carbonate [20], titanium dioxide [22,23], and aluminum hydroxide-oxide [24]. Microencapsulation technologies in inorganic materials mostly employ sol–gel techniques, starting from an oil-in-water (O/W) emulsion and alkoxysilane precursors [25,26,27]. For silica-based capsules, the most common precursor is tetraethyl orthosilicate (TEOS) [21,28,29], but the resulting shell is often too brittle and is easily subjected to damages and cracks [30]. A valid alternative is methyltriethoxysilane (MTES), which leads to the formation of an organosilica network. Chen et al. [30] synthesized paraffin microcapsules with an organosilica shell starting from an O/W emulsion (with commercial paraffin as the oil phase) and an MTES solution. The resulting capsules had an average diameter of 40–60 µm and a phase change enthalpy of 50–80% of that of pure paraffin, according to the initial relative amount of paraffin and MTES. A similar procedure was adopted by Tang et al. [31], who encapsulated lower molecular weight paraffin (octadecane) and obtained microcapsules with a diameter of 0.5–2 µm. Lin et al. [12] encapsulated stearic acid in organosilica shells, and graphene oxide was added to enhance the thermal conductivity and improve the encapsulation efficiency. 

Although these studies evidence the potentialities of sol–gel encapsulation and consider a quite broad range of PCMs, to the best of the author’s knowledge, only one study has been reported on the production of organosilica microcapsules with docosane as the PCM [27], which specifically refers to the encapsulation of n-docosane in ZnO/SiO_2_ shells prepared from TEOS. Docosane features a high phase change enthalpy and a melting/crystallization temperature of 41/33 °C, which makes it suitable for a wide range of applications, including thermal regulating fabrics, passive cooling systems for electronic devices, solar space heating materials and other solar thermal energy applications [32,33,34]. Moreover, although the reported research investigates the microstructural properties of the microcapsules to some extent, no studies have been found that use powerful techniques, such as the nuclear magnetic resonance (NMR), to deeply examine the phase change behavior in a confined volume, and relate the results to the outcome of microstructural and thermal analyses, such as X-ray diffraction (XRD) and differential scanning calorimetry (DSC). 

In most of the applications that need heat storage/management, the TES system is just an additional component that does not perform any other functions. However, this design approach increases weight and volume, which can be unacceptable for applications like automotive or portable electronics. This issue can be overcome by embedding the TES function directly in the structure of the device, with the help of multifunctional materials that can carry mechanical load and store/release thermal energy simultaneously. Such lightweight multifunctional materials could be polymer-matrix composites, as they combine the properties of different discontinuous phases embedded within a lightweight matrix [35]. Little has been done so far to develop and characterize such structural TES composites. Recently, our group prepared multifunctional epoxy/carbon laminates comprising carbon nanotubes-stabilized paraffin (CNTs) [36,37], polyamide/glass laminates with a microencapsulated and a shape-stabilized PCM [38,39], PCM-enhanced laminates starting from a novel reactive thermoplastic resin [40], and two types of semi-structural short carbon fibers composites including paraffin microcapsules, based on a thermoplastic (polyamide 12) [41] or a thermosetting (epoxy) [42] matrix, respectively.

Therefore, the aim of this research work is twofold. The first goal is to identify an effective sol–gel route to encapsulate docosane within organosilica shells of various dimensions and to deeply investigate how the confinement influences the microstructural and phase change properties of the PCM with a broad range of characterization techniques. The second purpose is to embed the prepared microcapsules in an epoxy/carbon laminate to produce a multifunctional structural TES composite, and to characterize the effect of the microcapsules on the mechanical properties of both the matrix and the laminate, and the overall TES capability of the system.

## 2. Materials and Methods

### 2.1. Materials

To prepare PCM microcapsules, n-docosane CH_3_(CH_2_)_20_CH_3_, (purity ≥ 98.5%), cetyltrimethylammonium bromide (CTAB) and absolute ethanol (purity ≥ 99.8%) were purchased from Sigma-Aldrich (Saint Louis, MO, US), and methyltriethoxysilane (MTES) (purity ≥ 98%) was provided by ABCR GmbH (Karlsruhe, Germany). Distilled water was used throughout all the process. All the materials were used as received, without further purification.

The prepared microcapsules were then embedded in multifunctional laminates, composed of a bi-component epoxy resin and a carbon fiber fabric. The epoxy resin Elan-tech^®^ EC 157 and the hardener Elan-tech^®^ W 342 were kindly provided by Elantas Europe S.r.l. (Collecchio (PR), Italy). Balanced plain weave carbon fiber (CF) fabric GG200P (mass per unit area = 192 g/m^2^, 3000 fibers per tow, linear density 200 tex) was purchased from G.Angeloni S.r.l (Quarto d’Altino (VE), Italy).

### 2.2. Preparation of the Microcapsules

**Figure 1:**
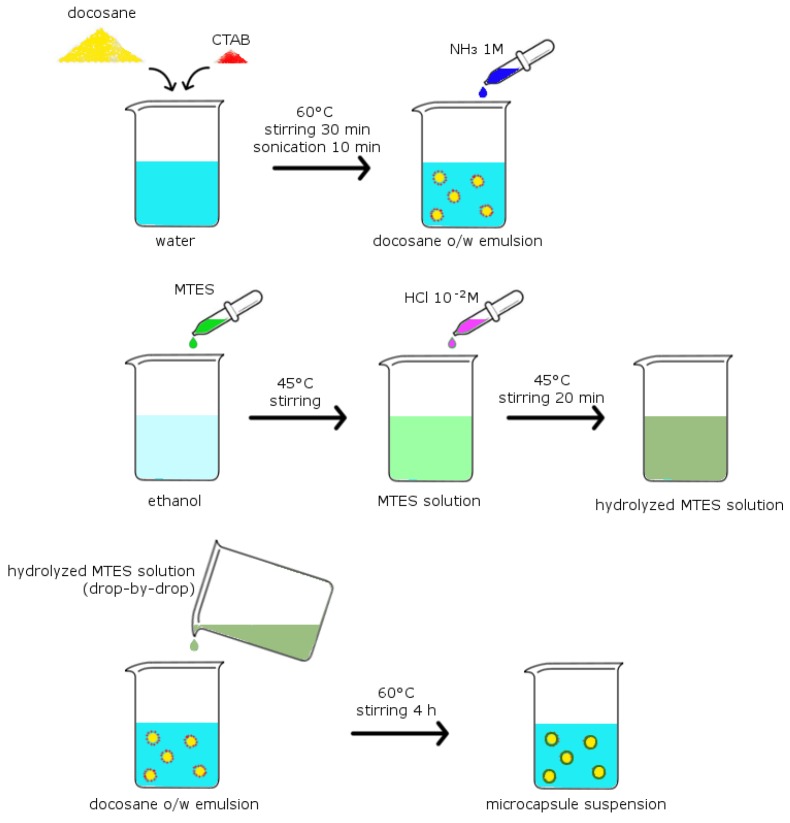
Experimental steps followed for the synthesis of the microcapsules.

Docosane-organosilica microcapsules were prepared with a protocol based on that reported in [31], modifying some key synthesis parameters in order to improve the emulsion stability and to obtain capsules with remarkably different sizes. The docosane o/w emulsion was prepared by adding 4 g of docosane and 0.05 g of CTAB to 50 mL water. The mixture was stirred for 30 min at 2500 rpm via a Dispermat F1 laboratory dissolver (VMA-Getzmann GmbH, Reichshof, Germany), and then sonicated for 10 min with a UP400S ultrasonic processor (Hielscher GmbH, Teltow, Germany). During stirring and sonication, the temperature was kept at 60 °C, above the melting point of the docosane. After sonication, the pH was adjusted to 8–9 by adding NH_3(aq)_ 1 M solution. The MTES solution was prepared in a different beaker, by adding 4.2 mL of MTES to ethanol at 45 °C under stirring. Then, 6.7 mL of HCl_(aq)_ 10^−2^ M was added drop by drop to promote the acid-catalyzed hydrolysis of MTES, and the solution was magnetically stirred at 45 °C for 20 minutes at 350 rpm. The MTES solution was then dropped into the docosane emulsion, and the total mixture was magnetically stirred at 60 °C at 350 rpm for 4 h. Finally, the suspension was filtered, and the filtrate was washed with hot water and ethanol (to remove free docosane and unreacted species) and dried overnight in a vacuum oven at 80 °C. The MTES solution was prepared with two different volumes of ethanol, i.e., 5.1 mL and 25.5 mL respectively, to modify the polarity of the synthesis medium and change the micelle size in the final suspension (MC1 and MC2). Each synthesis yielded approximately 2.5 g of microcapsules. Neat organosilica microparticles (Si) were also prepared without docosane and CTAB, under the same hydrolysis-condensation conditions [31,43]. The masses of the reagents for the three different preparations are reported in Table 1, while Figure 1 reports a schematic overview of the experimental protocol.

### 2.3. Preparation of the Epoxy-Based Matrices and Laminates

The microcapsules denoted as MC2 were employed to prepare multifunctional laminates, with an epoxy matrix and a carbon fiber fabric. 2.5 g of dry microcapsules were dispersed in 17.5 g of epoxy base, and the mixture was mechanically stirred to obtain a homogeneous dispersion. After that, 5.2 g of hardener was added to the mixture, to reach the base:hardener ratio of 100:30, as indicated on the resin technical data sheet. The nominal capsule mass fraction was 10 wt%. The mixture was then degassed and cast in silicon molds to prepare epoxy/MC samples for the subsequent characterization. The samples were cured for 24 h at room temperature and 10 h at 100 °C. Specimens with neat epoxy were also prepared for comparison. The same epoxy/MC2 mixture was used as a matrix to prepare 5-ply laminates with a bi-directional CF fabric, with a traditional hand layup technique. The resulting laminates had in-plane dimensions of 100 × 70 mm^2^. The laminates were vacuum bagged for 24 h at room temperature and post-cured at 100 °C for 10 h. Neat epoxy/CF laminates were prepared for comparison. The labeling adopted for the prepared epoxy-based samples is reported in Table 2.

### 2.4. Characterization of the Microcapsules

The morphology of the microcapsules was investigated through a field-emission scanning electron microscope (FE-SEM) Zeiss Supra 60 (Zeiss, Oberkochen, Germany) operating in high vacuum mode, after Pt-Pd sputtering at 30 k and 70 k magnifications. 

Fourier-transformed infrared spectroscopy (FTIR) was performed in attenuated total reflectance (ATR) mode with a Perkin Elmer Spectrum One instrument (Perkin Elmer, Waltham, MA, US). Data were collected in the wavenumber interval between 650 and 4000 cm^−1^, and four scans were superimposed for each spectrum (resolution 4 cm^−1^).

X-ray diffraction (XRD) measurements were collected by means of a Rigaku D-Max III powder diffractometer (Rigaku, Tokyo, Japan) using Cu-Kα radiation (λ = 0.154056 nm) and a graphite monochromator in the diffracted beam. A θ–2θ Bragg–Brentano configuration was adopted with the following scan conditions: scan range: 5–50° (in 2θ); sampling interval and counting time: 0.025° and 2 s, respectively. 

Nuclear magnetic resonance (NMR) has been used to further investigate the structure and molecular dynamics of the microcapsules. Solid-state NMR analyses were carried out with a Bruker 400WB spectrometer (Bruker, Billerica, MA, US) operating at a proton frequency of 400.13 MHz. ^13^C and ^29^Si cross-polarization (CP) magic angle spinning (MAS) spectra and proton-decoupled MAS spectra were collected. Spectra with CP pulse sequences were acquired under the following conditions: ^13^C frequency: 100.48 MHz, π/2 pulse: 3.5 µs, decoupling length: 5.9 µs, recycle delay: 4 s, 512 scans; contact time: 2 ms. ^29^Si frequency: 79.48 MHz, contact time: 5 ms, decoupling length: 6.3 µs, recycle delay: 20 s, 2k scans. Quantitative and relaxation experiments are detailed in the Supplementary Materials. Samples were packed in 4 mm zirconia rotor and spun at 8 kHz under air flow. Adamantane and Q_8_M_8_ were used as external secondary references. Si units were labeled according to the usual NMR notation, T^n^ representing trifunctional Si units with n bridging O atoms. 

Differential scanning calorimetry (DSC) was performed to study the variation in the phase transition temperature and enthalpy values of docosane in its bulk and encapsulated state. Specimens of approx. 8 mg were sealed in aluminum crucibles and tested in a Mettler DSC 30 calorimeter (Mettler Toledo, Columbus, OH, USA), at 10 °C/min, between 0 and 70 °C, under a nitrogen flow of 100 mL/min. The employed cooling medium was liquid nitrogen. A heating scan, a cooling scan, and a second heating scan were performed for each specimen. The test allowed the measurement of the melting and crystallization temperatures ($T_{m}$, $T_{c}$) and enthalpy values ($\Delta H_{m}$, $\Delta H_{c}$,) of the PCM phase. The encapsulation efficiency η was evaluated for each sample as the ratio between its phase change enthalpy and that of the neat docosane, through Equation (1), as

(1) $$\eta =\frac{\Delta H_{m}^{MC}+\Delta H_{c}^{MC}}{\Delta H_{m}^{D}+\Delta H_{c}^{D}},$$

where $\Delta H_{m}^{MC}$ and $\Delta H_{c}^{MC}$ are the melting and crystallization enthalpy values of the microcapsules and $\Delta H_{m}^{D}$ and $\Delta H_{c}^{D}$ are those of the neat docosane.

Moreover, a kinetic analysis was performed on neat docosane and MC1 to investigate the effect of encapsulation on the activation energy of the phase change. Tests were performed a 10 °C/min, 1 °C/min and 0.2 °C/min. The activation energy $E_{a}$ was determined as the slope of the linear regression, through a standard Arrhenius approach, as reported in Equation (2):

(2) dln(ϑ)d(1Tp)=−EaR,

where *ϑ* is the heating or cooling rate, $T_{p}$ is the peak phase change temperature (in K) and R is the universal gas constant, equal to 8.314 J/mol·K.

Lastly, thermogravimetric analysis (TGA) was performed to study the thermal stability of the docosane and the microcapsules. The tests were performed on a Mettler TG50 instrument (Mettler Toledo, Columbus, OH, USA). Specimens of approx. 15 mg were tested at 10 °C/min up to 700 °C, under a nitrogen flow of 100 mL/min. The test allowed the measurement of the temperatures corresponding to a mass loss of 1 wt% ($T_{1\%}$), 3 wt% ($T_{3\%}$), and 5 wt% ($T_{5\%}$), as well as the peak temperatures of the mass loss derivative signal, corresponding to the maximum degradation rate of the docosane ($T_{p}^{D}$) and organosilica ($T_{p}^{Si}$) phases. Moreover, the weight fraction of docosane ($\omega_{D}^{TGA}$) could be estimated for each sample from the mass loss after the degradation of this phase, and the residual mass after the test ($R_{700^{\circ }C}$) was also measured.

### 2.5. Characterization of the Epoxy-Based Matrices and Laminates

For the microstructural analysis, specimens of matrices and laminates were cryogenically fractured in liquid nitrogen, and the fracture surface was observed with the FE-SEM Zeiss Supra 60 scanning electron microscope (Zeiss, Oberkochen, Germany), in high vacuum mode, after Pt-Pd sputtering. 

DSC and TGA analyses were performed with the same procedure described in the previous paragraph for the characterization of the microcapsules. From the DSC tests, an experimental content of docosane in each sample was calculated with Equation (3), as

(3) $$\omega_{D}^{DSC}\left(wt\%\right)=\frac{\Delta H_{m}}{\Delta H_{m}^{D}},$$

where $\Delta H_{m}$ is the experimental melting enthalpy measured on each epoxy-based sample. Moreover, the microcapsule weight fraction was experimentally determined according to Equation (4), as

(4) $$\omega_{MC2}^{DSC}\left(wt\%\right)=\frac{\Delta H_{m}}{\Delta H_{m}^{MC2}},$$

where $\Delta H_{m}^{MC2}$ is the experimental melting enthalpy of the microcapsules MC2. These results were then compared with those obtained in TGA tests. 

The TGA analyses lead to the measurement of the degradation temperatures $T_{1\%}$, $T_{3\%}$ and $T_{5\%}$, as reported for the characterization of the microcapsules, as well as the peak temperatures of the mass loss derivative signal, corresponding to the maximum degradation rate of the docosane ($T_{p}^{D}$) and epoxy ($T_{p}^{EP}$) phases. Moreover, the fiber weight fraction could be calculated from the residual masses at the end of the test, also by considering the residual masses of EP and EP-MC2 matrices. From these results, a theoretical density was calculated and compared with the experimental density obtained via the Archimedes’ balance technique weighing the samples in ethanol ($\rho_{EtOH}$ = 0.80458 g/cm^3^, measured at 20 °C) with a Gibertini E42 analytical balance (Gibertini, Novate Milanese (MI), Italy). This comparison allowed the calculation of the volume fraction of pores. Additionally, from the TGA tests, the degraded mass at 260 °C (i.e., immediately after the degradation of the docosane phase) was employed to determine an experimental weight fraction of docosane, which was compared with that obtained with DSC measurements.

Three-point flexural tests were performed according to ASTM D790-03 standard with an electromechanical dynamometer Instron 5969 (Instron, Norwood, MA, USA), equipped with a 50 kN load cell. Three specimens were tested for each sample. For samples EP and EP-MC, the test was performed at a crosshead speed of 1.5 mm/min, on casted specimens with nominal dimensions of 70 × 10 × 3 mm^3^, which were tested flatwise with a span length of 50 mm. The tangent modulus of elasticity (E), the flexural strength ($\sigma_{fM})$ and the flexural strain at break ($\varepsilon_{b})$ were determined with Equations (5)–(7), as

(5) $$E=L^{3}m/4bd^{3},$$

(6) $$\sigma_{fM}=3PL/2bd^{2},$$

(7) $$\varepsilon_{b}=6Dd/L^{2},$$

where *L* is the support span, m is the slope of the tangent to the initial portion of the load-deflection curve, *b* and *d* are the specimen width and thickness, *P* is the maximum load and *D* is the deflection at the break point. For the samples EP-CF and EP-MC-CF, specimens with nominal dimensions of 70 × 10 × 3 mm^3^ were cut from the prepared laminates by a diamond wheel and tested flatwise with a span length of 70 mm and at a crosshead speed of 9 mm/min. The values of E and $\varepsilon_{b}$ were determined with Equations (5) and (7), respectively, but the value of $\sigma_{fM}$ was determined with the Equation (8), as

(8) $$\sigma_{fM}=\frac{3PL}{2bd^{2}}\left[1+6{\left(\frac{D}{L}\right)}^{2}-4\left(\frac{D}{L}\right)\left(\frac{d}{L}\right)\right],$$

to consider the not negligible forces developed at the supports that come from the considerably high span-to-thickness ratio, necessary when the in-plane resistance is remarkably higher than the interlaminar resistance, as it is often true in case of laminates. 

A quick test was performed to check the overall thermal management performance of the laminates. Each laminate was heated in an oven for 30 min at 60 °C, above the melting temperature of docosane, then taken out and left cooling down to room temperature under laboratory conditions. During the cooling phase, the surface temperature was recorded with an infrared thermal imaging camera FLIR E60 (FLIR, Limbiate (MB), Italy), placed at approx. 30 cm from the sample. 

Dynamic mechanical thermal analysis (DMA) was performed on the laminates to investigate the effect of the phase transition of docosane on the viscoelastic properties. The tests were performed with a TA Q800DMA instrument (TA Instruments, New Castle, DE, US), on specimens with nominal in-plane dimensions 35 × 5 mm^2^ and the thickness of each laminate. The tests were performed in single cantilever mode, and the distance between the grips was fixed at 17.5 mm. Storage modulus and loss modulus were measured in the range −20–140 °C at 3 °C/min, with an applied strain of 0.05% at a frequency of 1 Hz.

## 3. Results and Discussion

### 3.1. Characterization of the PCM Microcapsules

#### 3.1.1. Sample Preparation and Quality of the Docosane o/w Emulsion

The first part of the activity was focused on optimizing the emulsion parameters to maximize the encapsulated docosane. Since an important factor is the choice of the surfactant, a first attempt was made with sodium dodecyl sulfate (SDS), employed in many research works to obtain o/w emulsions with different types of paraffins [12,13,21,22,23,28,30,31]. However, it was noticed that with docosane this surfactant is not highly effective and leads to low fractions of emulsified paraffin. This behavior could be related to the higher molecular weight of docosane with respect to the paraffins employed in other studies (e.g., octadecane, eicosane). Nevertheless, most of the considered works do not report information on the quality of the emulsion nor on the synthesis yield, related to the ratio of encapsulated paraffin with respect to the initial amount, and therefore it is difficult to make a direct comparison. It should be pointed out that the study of PCM emulsion stability is important not only to maximize the final encapsulation yield but also for the development of high performing heat transfer fluids [44,45,46], which often contain emulsified PCM. In the present study, considerably more effective emulsification of docosane was obtained with CTAB, probably due to its higher hydrophobic character denoted by its lower hydrophile-lipophile balance (HLB) number [26,47,48,49]. 

#### 3.1.2. SEM Micrography

**Figure 2:**
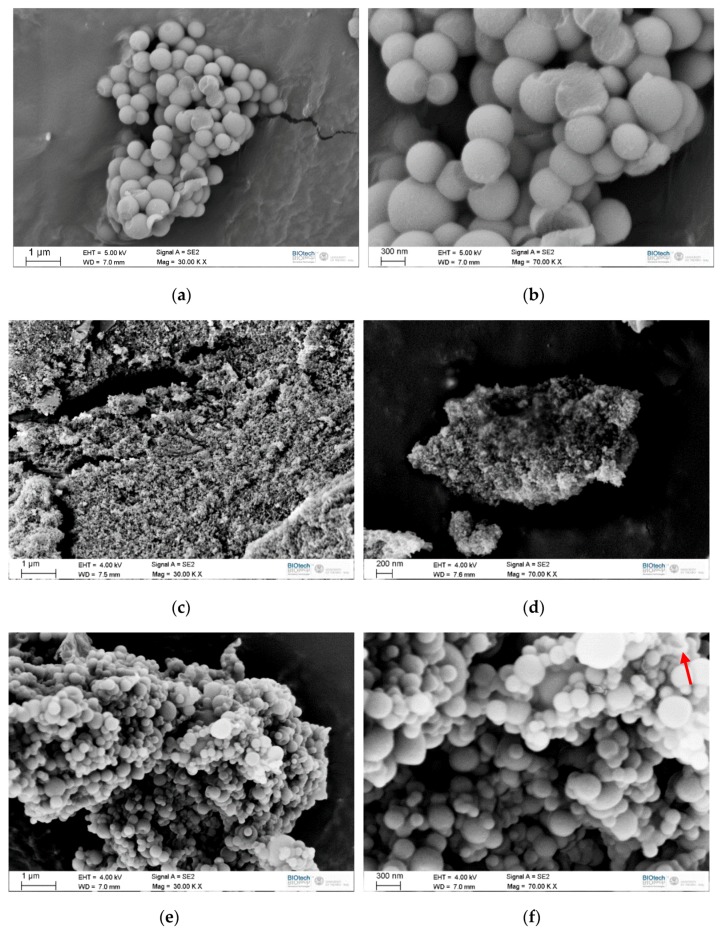
SEM micrographs of the prepared samples. (**a**,**b**) Si, (**c**,**d**) MC1, (**e**,**f**) MC2.

Figure 2a–f reports SEM micrographs of the prepared microcapsules.

The neat organosilica particles (Si, Figure 2a,b) present a spherical shape, a smooth surface, and dimensions of 606 ± 110 nm. They are monodispersed with a relatively narrow size distribution in agreement with previous results [32,34]. However, microparticles appear strongly aggregated, as clearly observable at higher magnification in Figure 2b. For the samples containing microencapsulated docosane, the first manifest difference between MC1 (Figure 2c,d) and MC2 (Figure 2e,f) is the particle size. The sample MC1 is composed of strongly aggregated and extremely small particles, with a diameter of approximately 50 nm. It was not possible to acquire images at higher magnifications, due to the instability of the sample under the electron beam during focusing, and thus the analysis of the particle size was performed manually on a digitally zoomed micrograph. Due to the blurriness of the resulting image, it was difficult to perform an accurate analysis, but the measured diameters fell in the range 35–60 nm. The particles of the sample MC2 are bigger, spherical, and with shape, surface roughness and state of aggregation resembling those of neat organosilica spheres. MC2 particles are smaller than Si ones (244 ± 98 nm) and have a higher coefficient of variation. The majority of the capsules are intact, but a core-shell morphology can be observed from the sporadically broken capsules, as indicated with a red arrow in Figure 2f.

#### 3.1.3. FTIR Spectroscopy on the Microcapsules

**Figure 3:**
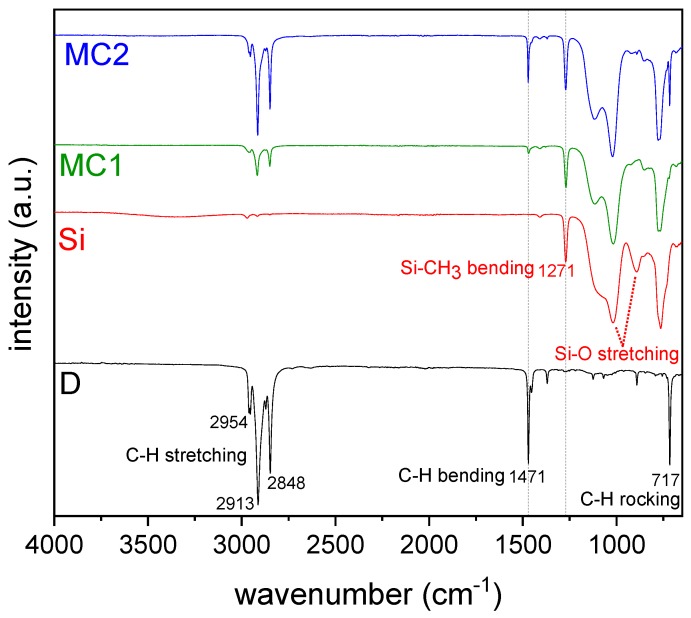
FTIR spectra of the bulk docosane (D), docosane-organosilica core-shell microparticles (MC1 and MC2) and the neat organosilica microparticles (Si).

Figure 3 reports the FTIR spectra obtained on the bulk docosane (D), MC1 and MC2 microcapsules and the neat organosilica microparticles (Si).

The spectrum of the neat docosane is characterized by the typical vibrations of methylene groups; the peaks at 2954, 2913, 2872 and 2848 cm^−1^ can be assigned to the C–H bond stretching vibration in –CH_2_ and –CH_3_ groups, while the peaks at 1471, 1454 and 1370 cm^-1^ can be attributed to the asymmetric and symmetric bending of –CH_2_ and –CH_3_ groups, respectively, and the peak at around 717 cm^−1^ is due to the rocking vibration of the –CH_2_ group [32,34]. On the other hand, the sample Si shows the peaks associated with the organosilica network originated from MTES. The small peaks in the interval 3000–2800 cm^−1^ are related to the stretching vibration of the C–H bond in the methyl group, and the peak at 1271 cm^−1^ is due to the bending vibration of the Si–CH_3_ bonds [50]. The two broad bands around 1117 and 1021 cm^−1^ and the weak signal at 924 cm^−1^ are due to the asymmetric stretching vibrations of siloxane bonds and silanols, respectively [51]. The peak at 853 cm^−1^ is due to Si–O symmetric stretching and the signal at 777 cm^−1^ can be attributed to the Si–C bond vibration [51]. Both docosane and organosilica signals are present with different relative intensity in the spectra of the two prepared microencapsulated samples, thus proving the presence of the paraffin core in MC1 and MC2. The peaks associated with docosane are more intense in MC2 than in MC1, indicating a higher docosane content in the larger microcapsules. The siloxane band appears narrower in comparison with Si sample, particularly in the case of MC2. From the FTIR analysis, it is possible to make some semi-quantitative considerations based on sharp peaks belonging to the same spectral region. By measuring the intensity ratio of the two peaks indicated with dotted lines in Figure 3, i.e., the signal at 1471 cm^−1^ related to methylene docosane, and the one at 1271 cm^−1^ attributed to Si–CH_3_ in organosilica, the docosane-to-organosilica molar ratios result of 0.15 and 0.55 for MC1 and MC2, respectively, in agreement with the results of NMR and TGA analyses reported below. 

#### 3.1.4. XRD Analysis

**Figure 4:**
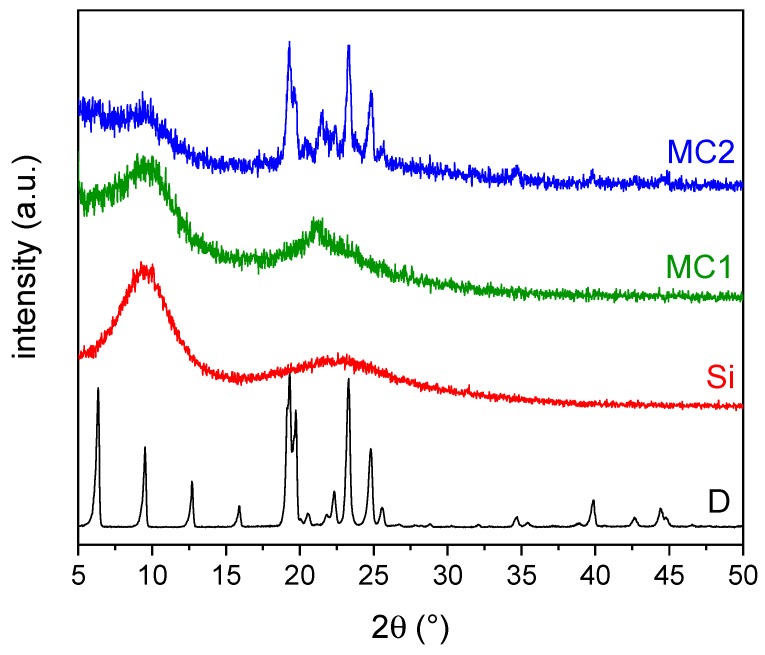
XRD spectra of the bulk docosane (D), MC1 and MC2 microcapsules and the neat organosilica microparticles (Si).

Figure 4 reports the XRD spectra of the samples D, MC1, MC2, and Si.

For the spectrum of the neat docosane, the signals can be attributed to the triclinic phase of solid docosane, stable up to approx. 40 °C [52,53]. This phase is characterized by the spatial group $P\bar{1}$, generally indicated as γ_0_(C22), with cell parameters a = 4.2805 Å, b = 4.8212 Å, c = 28.2877 Å, α = 91.14°, β = 94.63°, γ = 106.39°. On the other hand, the Si spectrum displays the typical diffraction pattern of the amorphous MTES-derived organosilica, with two broad halos located at around 10° and 23° [12,30,31]. 

The XRD spectrum of the microcapsules MC1 resembles that of the neat organosilica, and the diffraction pattern of the docosane phase is not detectable unless a broad and less intense peak centered at 21°. On the contrary, in the spectrum of MC2 both the broad halos of the organosilica phase and the sharp peaks of the docosane phase, especially between 18° and 28°, are clearly detectable, whereas the peaks at lower angles are not visible. In this spectrum, the docosane reflection at 21.4° is relatively more intense with respect to the spectrum of D. This may suggest the presence of a different solid crystallographic phase, the so-called rhombohedral rotator phase R-II, which in normal conditions is stable near the melting temperature [45]. This rotator phase is a crystalline mesostate with a rotational degree of freedom along the chain axis [19]. This result, therefore, suggests that the confinement of the paraffin chains inside the organosilica shells induces a structural disorder; in fact, the absence of (00l) peaks in MC2 spectrum, located at angles lower than 18°, can be attributed to the disappearance of the lamellar ordering of the γ_0_(C_22_) structure, whereas the lateral arrangements of the chains remain unchanged (peaks located at angles greater than 18°) [54]. On the other hand, the reduction of the particle size in MC1 sample, as revealed by SEM analysis, leads to a more pronounced distorted situation, and only the signal of the rotator state appears with a shift towards lower angle than for MC2 sample. This evidence suggests that the degree of confinement of docosane in organosilica microcapsules can be related to the particle size [54].

#### 3.1.5. Solid-State NMR Analysis

**Figure 5:**
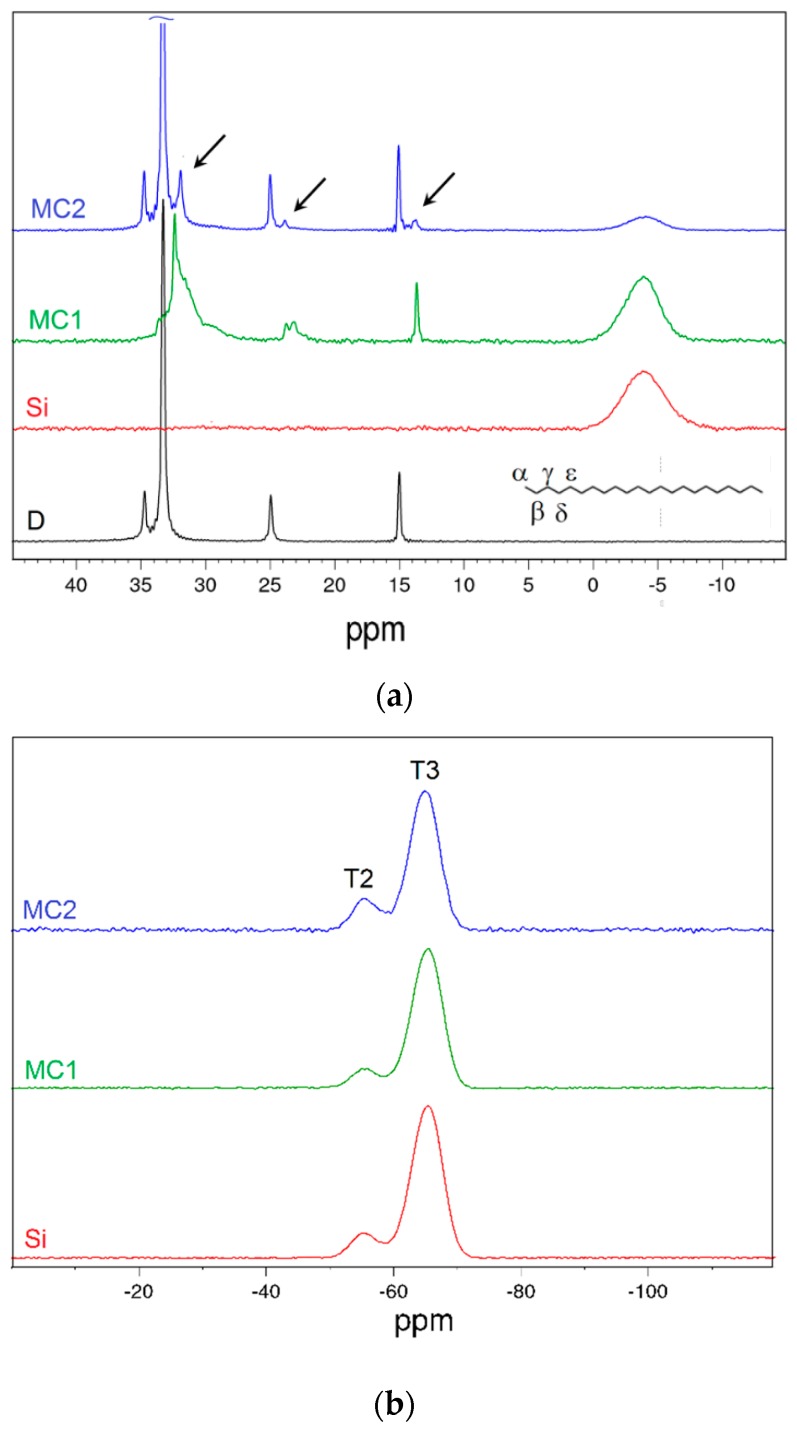
NMR spectra of docosane (D), MC1 and MC2 microcapsules and the neat organosilica microparticles (Si): (**a**) ^13^C CP-MAS spectra with docosane carbon labeling and arrows to highlight rotator phase signals; (**b**) ^29^Si CP-MAS spectra.

Solid-state NMR analysis was performed to deeply investigate the microstructure, the morphology and the confinement effect of the different microcapsules. Figure 5a,b shows the ^13^C and ^29^Si CP-MAS spectra of the neat docosane, the two microencapsulated PCMs, and the organosilica particles.

The spectrum of bulk docosane in Figure 5a shows the typical signals of the n-alkanes, associated with the first four carbon atoms in the chain (indicated as α, β, γ, δ in the structural scheme, Figure 5a), while the chemical shifts are not anymore resolvable from the fifth (ε) carbon atom on [55]. For the neat organosilica microcapsules (Si), the only signal is found at approx. −4 ppm and it is due to the methyl carbon atoms linked to silicon, while the absence of signals associable to the ethoxy groups indicates the completion of MTES hydrolysis [51]. Together with all the paraffin and MTES signals, the docosane phase in the sample MC2 shows also some minor upfield shifted peaks (approx. 2 ppm) close to the α, β and ε signals, indicated with arrows in Figure 5a, which can be associated with α, β and ε methylene groups in a different environment. Interestingly, in the spectrum of MC1, the chemical shift of docosane signals fits with the weak upfield resonances of MC2 spectrum, and there are no signals in the original positions. The upfield shifted resonances can be due to the presence of the rotator phase, already mentioned in paragraph 3.1.4, or also to the γ-gauche effect near the chain ends, which causes a non-uniform distribution of the conformational disorder, thereby broadening the peaks [56]. This behavior could be attributed to the confinement effect of the docosane inside the capsules, and the fact that it is more evident for the smaller (MC1) capsules supports this hypothesis [57] in agreement with XRD conclusions. In this sense, it could be useful to evaluate the signals of the ^13^C proton-decoupled MAS spectrum (Figure S1 and related discussion, see Supplementary Materials). As the spin-lattice relaxation times of the various carbon atoms are remarkably different from each other (e.g., for α-C it is 1.2 s, for β-C 16 s, for γ-C and ε-C >700 s), with the selected experimental parameters it is possible to make a quantitative comparison among the methyl groups. From the ratio between the areas of H_3_C–Si (−4 ppm) and H_3_C–CH_2_ (14 ppm) signals, the molar ratio organosilica/docosane can be deduced, considering that the signal at −4 ppm counts for one carbon atom and that at 14 ppm for two. The results of this calculation give docosane-to-organosilica molar ratios of 0.09 and 0.53 for MC1 and MC2, respectively. What is immediately evident is that for the sample MC1 the silica fraction is approx. 6 times higher than for MC2, which is in good agreement with both the ratios calculated from FTIR peaks (Section 3.1.3) and the DSC results (Section 3.1.6). Moreover, by integrating the area of α and α’ signals, it is possible to estimate that the rotator phase (represented by α’) is the 20% of all the encapsulated alkane for sample MC2, while for the MC1 it represents the 100%. 

Furthermore, through molecular dynamics NMR experiments (see Figure S2 in Supplementary Materials for details), and especially from the evaluation of ^13^C spin-lattice relaxation times (T1_C_), it is possible to prove that the two detected phases represent respectively a free bulk paraffin fraction and a docosane fraction interacting with the inner silica shell, since the interaction causes a remarkable reduction of T1_C_. A similar effect was already observed by Inoue et al. [58], who studied the behavior of polyethylene on silica surfaces and imputed the constraints of the molecular motions of the polymer chains to the interaction with the silica surface. As evidenced by the XRD measurements, the inclusion can cause an alteration of the crystalline structure. This effect was already mentioned by Okazaky [56], who studied through T1 analysis the different behavior of even and odd alkanes. As a matter of fact, despite the very long T1_C_ of main chain carbons of pure docosane measured by Okazaky, the present samples show a reduced and capsule size-dependent T1 value for main chain methylenes, whereas it is practically unchanged for methyls (Table S1). No effects due to interaction can be detected for silica methyls, as also showed by Okazaky’s samples. The overall size-dependent T1_C_ reduction is a proof of the effective paraffin inclusion and the lower values shown by α’ and β’ with respect to α and β further indicate the interaction of a docosane fraction with the silica shell, which leads to the alteration of the crystal structure in perfect agreement with XRD conclusions. 

Finally, Figure 5b shows the ^29^Si CP-MAS of the samples MC1, MC2, and Si, to investigate the degree of condensation of the organosilica phase. All the samples show the signals of the T^2^ (R-Si(OSi)_2_(OH)) and T^3^ (R-Si(OSi)_3_) units, at −55.7 ppm and −65.6 ppm, respectively [51], and the intensity ratios are comparable in the three cases, which implies that the presence of docosane and CTAB surfactant does not influence MTES hydrolysis-condensation process. The quantitative analysis was performed on the ^29^Si proton-decoupled MAS spectra (not reported) and confirms the trends observed in the CP-MAS spectra, being the condensation levels approx. 96% for the sample Si and 94% for the sample MC2. 

#### 3.1.6. DSC Analysis of the Microcapsules

**Figure 6:**
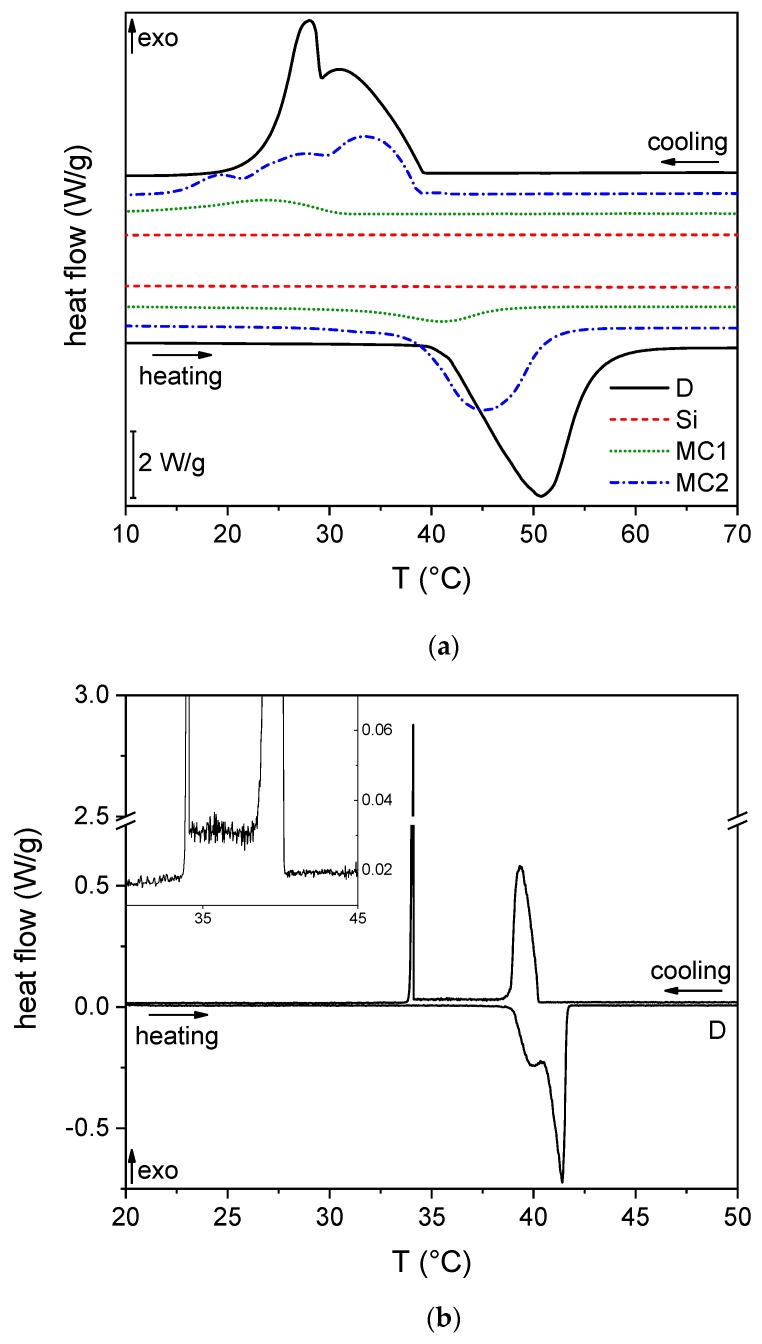

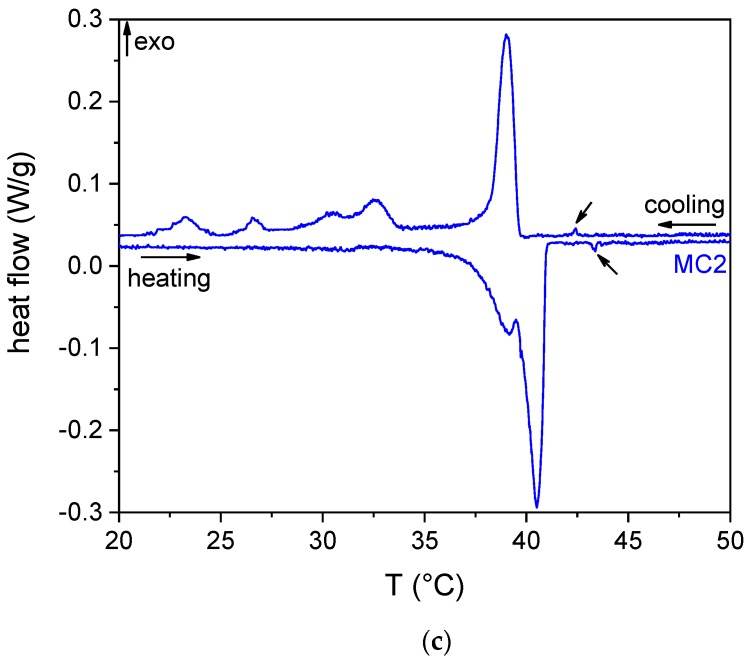
(**a**) DSC thermograms of the bulk (D) and microencapsulated (MC1 and MC2) docosane and the neat organosilica microparticles (Si). The first heating scans and the cooling scans are reported. (**b**) DSC thermogram of the neat docosane acquired at 0.2 °C/min (heating and cooling scan). (**c**) DSC thermogram of the microcapsules MC2 acquired at 0.2 °C/min (heating and cooling scan).

Figure 6a shows the DSC thermograms of the samples Si, D, MC1, and MC2. The neat organosilica does not manifest any thermal transition in the considered temperature interval. On the other hand, in the heating scan, all the samples containing docosane experience the melting phase change, visible as a single broad peak at the adopted heating scan (10 °C/min). 

The data reported in Table 3 show that the peak melting temperature ($T_{m}$) measured on the microcapsules is lower than that of the neat docosane, and this is more evident for the smaller (MC1) than for the bigger (MC2) capsules. This effect, also reported by other literature studies [29,30,31,33,59,60,61], can be ascribed to the fact that the confinement in a small volume hinders the crystallization, but in this specific case also to the interaction of docosane with the inner shell surface, which limits the chain mobility as evidenced by NMR studies (see Section 3.1.5). This hypothesis is supported by the reduction in the melting enthalpy, which is more evident for the smaller capsules presenting a higher surface-to-volume ratio. The same effect is at the basis of the reduction in the peak crystallization temperature for the sample MC1; as the paraffin domains are confined in smaller volumes, a higher supercooling degree is needed to initiate the crystallization. This phenomenon must be taken into account when designing a microencapsulated PCM, as it could change its application temperature interval. The cooling scans are characterized by the presence of several peaks. The neat docosane shows two peaks, the first (33.5 °C) related to the liquid-solid phase change, and the second (28.5 °C) associated with a solid–solid transition [62], while the microcapsules show several peaks, visible especially in the sample MC2, which can be due to confinement effect and the presence of different crystalline phases. The measured phase change enthalpies are reported in Table 3. The neat docosane develops 234.2 J/g, and the melting enthalpies measured on MC1 and MC2 provide information about the docosane fraction in each sample, described by the efficiency (η) reported in Table 3. The sample MC2 contains approx. 60% of docosane, while the PCM fraction decreases to 14% for MC1. These data are in good agreement with the NMR (see Section 3.1.5) and TGA (see Section 3.1.7).

Figure 6b,c reports the DSC thermograms of D and MC2 acquired at 0.2 °C/min. As the lower heating rate allows a better resolution of the signals, the neat docosane (Figure 6b) shows two endothermic peaks in the heating scan, one (at lower temperature) associated with the solid–solid transition from the crystalline phase to the rotator phase, and the other due to the solid-liquid phase transition, in good agreement with the detailed investigation performed by Wang et al. [62]. The cooling scan shows two sharp peaks with a broad halo in between, which is better visible from the inset plot. Following Wang’s analysis, the peak at higher temperature can be attributed to the transition from the liquid state to the rotator phase, the broad halo is related to the rotational action of CH_2_ bonds, and the last sharp peak due to the last solid–solid transition, which ends with the formation of an ordered crystalline phase. The encapsulated docosane MC2 shows the same two peaks as the sample D in the heating scan, but at a slightly lower temperature, and at least five intense peaks in the cooling scan. This implies a different crystallization behavior due to confinement and interaction with the organosilica shell. A very small peak (indicated with arrows on Figure 6c) can also be observed both on heating and on cooling. This is probably due to the surface freezing phenomenon, i.e., to the fusion/crystallization of a monolayer formed on the surface of liquid docosane, observable here due to the high surface-to-volume ratio of the microencapsulated docosane and not normally observable on bulk samples [62].

The DSC tests at different heating scans were also performed to determine the apparent activation energy ($E_{a}$) of the phase transition of the bulk and microencapsulated docosane. The results are reported in Figure S3 in Supplementary Materials. The value of $E_{a}$ for docosane melting is 501 kJ/mol, in good agreement with similar systems [63]. Despite the not negligible error bands, related to the values of R2 lower than 1 in the linear regressions of the phase change temperatures, it can be appreciated that the values of $E_{a}$ increase upon encapsulation, probably due to steric effects and interaction with the capsule shell.

#### 3.1.7. TGA Analysis of the Microcapsules

**Figure 7:**
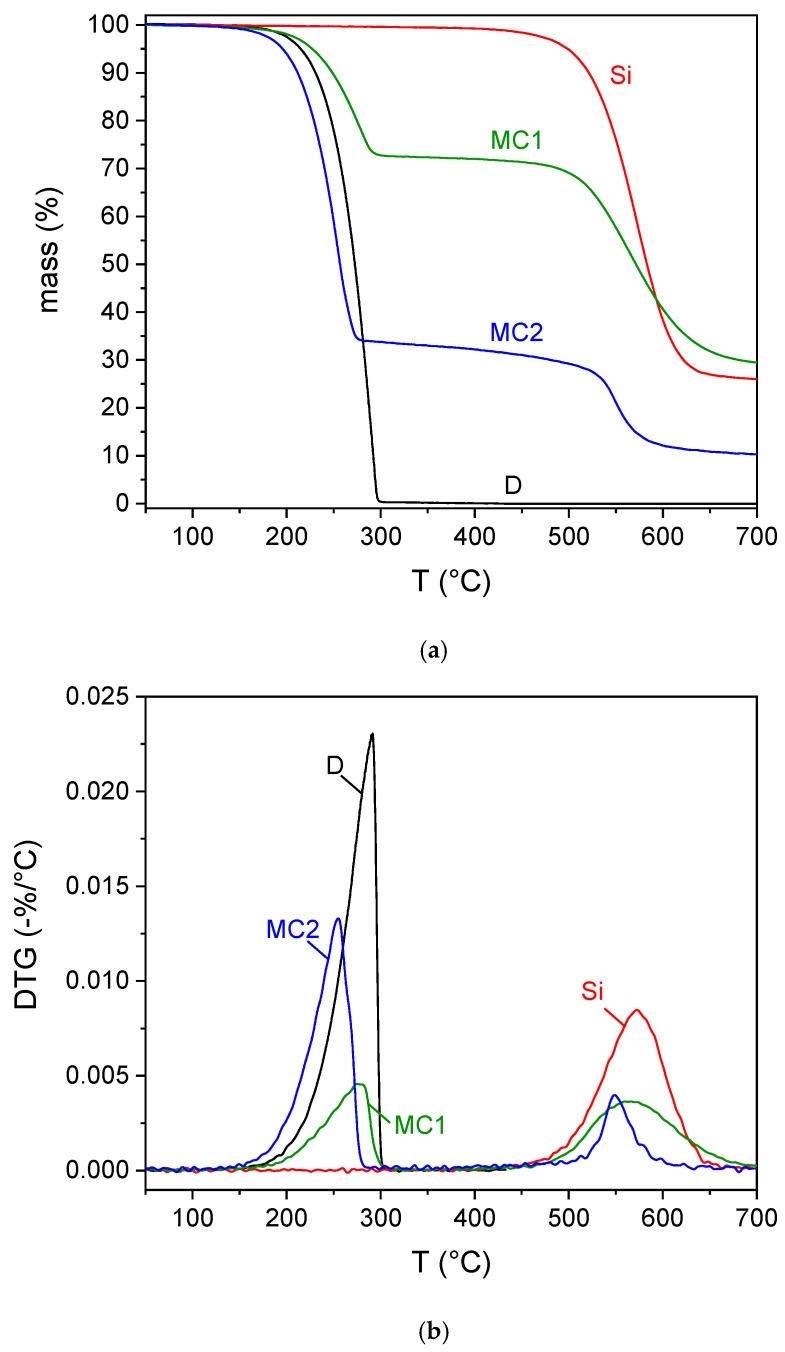
TGA thermograms of the bulk (D) and microencapsulated (MC1 and MC2) docosane and the neat organosilica microparticles (Si). (**a**) mass; (**b**) mass loss derivative (DTG).

Figure 7a,b shows the TGA thermograms on the prepared microcapsules, while the most important results are displayed in Table S2. 

The bulk docosane undergoes a single-step thermal degradation with $T_{p}^{D}$ at 291 °C, while the neat organosilica is thermally stable until approx. 500 °C. Both the degradation steps are present in the samples MC1 and MC2, but the derivative peak temperatures are of some degrees lower than those of the neat samples, which was already reported in the literature, especially for the PCM phase [31]. From the amplitude of the degradation step of docosane, it is possible to estimate the docosane weight fraction for the samples MC1 and MC2, which are reported in Table S2. The fraction of docosane calculated for MC2 is 65%, and this is in good agreement with the DSC results, while that determined for MC1, 26%, is slightly higher than that calculated via DSC, and this can be due to sample inhomogeneity, but also to the fact that the crystallinity degree of the docosane encapsulated in small microcapsules, such as the sample MC1, is lower than that of the free docosane, and thus the developed enthalpy is lower than expected, which is in agreement with the XRD results. It is worth noting that the docosane weight content calculated by TGA is in good agreement with the molar amount estimated by both FTIR and NMR analyses.

### 3.2. Characterization of the Epoxy-Based Matrices and Laminates

The characterization previously described highlights the higher melting enthalpy and the overall better performance of the capsules MC2. Therefore, this PCM was used in combination with an epoxy matrix and continuous carbon fibers to produce multifunctional composites. An extensive characterization was performed to assess the influence of the microcapsules on the properties of the neat epoxy and the epoxy/carbon laminate. The results of this characterization are discussed hereafter.

#### 3.2.1. SEM Microscopy on the Matrices and Laminates

**Figure 8:**
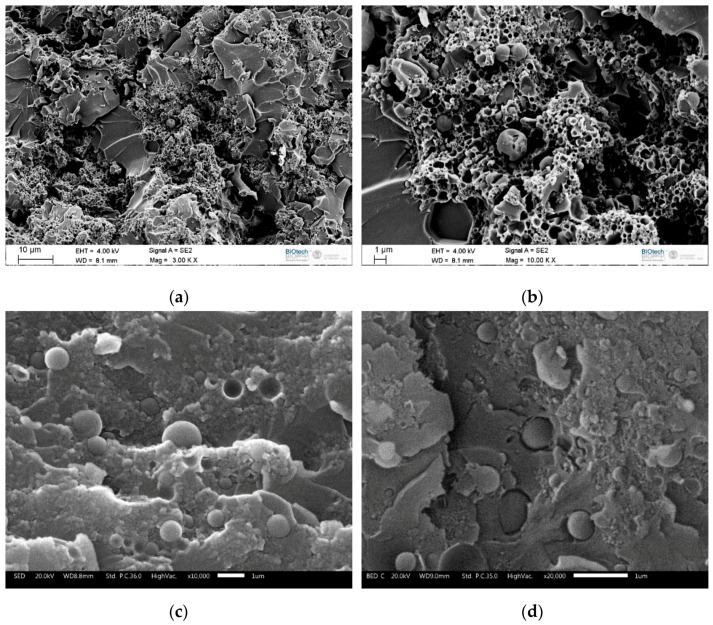
SEM micrographs of the samples EP-MC2 (**a**,**b**) and EP-MC2-CF (**c**,**d**).

Figure 8a–d shows the SEM micrographs of the cryofracture surface of the EP-MC2 matrix and EP-MC2-CF composite.

The micrographs of the sample EP-MC2 show the presence of aggregates and agglomerates, which have already been observed in the micrographs of the neat particles (see Figure 2). Although the state of aggregation of PCM micro- or nano-capsules is a very important parameter, it is not commonly described in the research articles dealing with the synthesis of microencapsulated PCM, and therefore the results of this study can hardly be compared to previous work. On the other hand, agglomerates in the composites could be avoided by improving the mixing conditions. Although the attempts made so far, which included solvent-assisted dispersion, have not been successful, further effort will be made to improve the dispersion of the microcapsules in the epoxy matrix. It can also be noticed that the capsule-matrix adhesion is not excellent, as the fracture is adhesive and occurs at the interface. The use of another silane precursor instead of MTES, with a different functional group (e.g., aminopropyltriethoxysilane, APTES), could be beneficial to overcome this problem and improve the chemical compatibility between the epoxy and the capsule shells. The same conclusions can be made for the micrographs of the composites, which focus on the capsule-rich interlaminar region.

#### 3.2.2. DSC on the Matrices and Laminates

**Figure 9:**
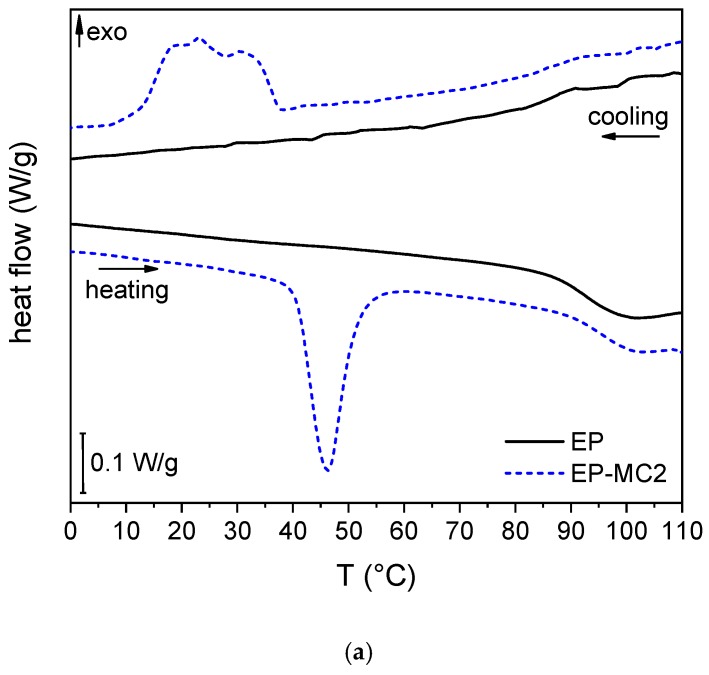

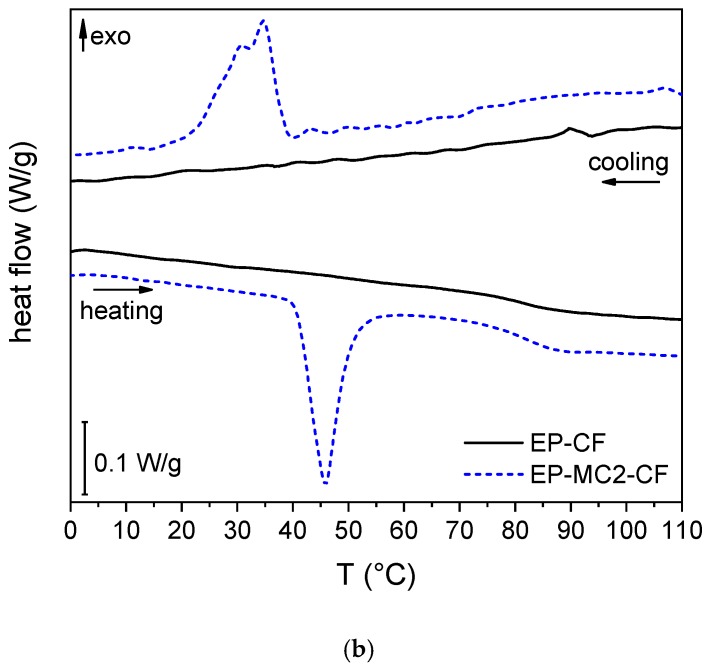
DSC thermograms of the prepared epoxy-based matrices and laminates. The first heating scans and the cooling scans are reported. (**a**) EP and EP-MC2; (**b**) EP-CF and EP-MC2-CF.

The DSC thermograms of the epoxy matrix with and without microcapsules (EP and EP-MC2) are reported in Figure 9a. The sample EP presents a single glass transition at approx. 94 °C, while the thermogram of the sample EP-MC2 also shows the peaks associated with the phase change of the microencapsulated docosane. As shown in Table S3, the developed phase change enthalpy is approx. 14 J/g, and from this value the experimental weight fraction of docosane and MC2 can be estimated as 6.2 wt% and 10.2 wt%, respectively, which matches the nominal weight fraction of MC2 (10 wt%) and the fraction of docosane in the MC2 microcapsules (approx. 60 wt%). This implies that the TES properties of the microcapsules are also preserved after the production process needed to embed them in an epoxy matrix. The same analysis was conducted on the carbon fiber laminates, whose DSC thermograms are reported in Figure 9b. Once again, the PCM-filled sample shows peaks related to the phase transition of the filler, and an experimental mass fraction of docosane of 4.1 wt% can be calculated from the developed enthalpy. As reported in Table S3, the melting and crystallization peak temperatures of the laminate are lower and higher, respectively, than those of the sample EP-MC2, which can be due to the higher thermal conductivity induced by the carbon fibers.

#### 3.2.3. TGA Analysis and Fraction of the Constituents

**Figure 10:**
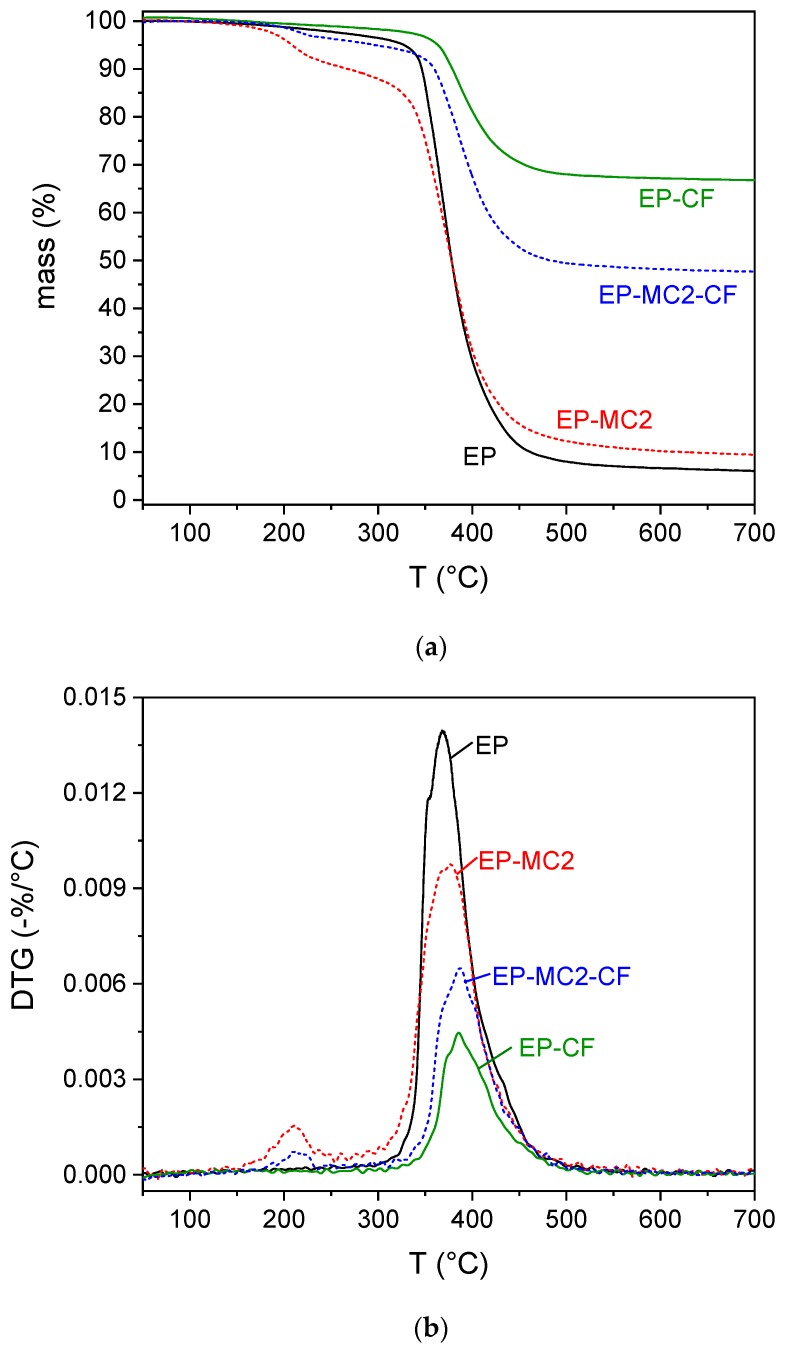
TGA thermograms of the epoxy-based matrices EP and EP-MC2, and on the laminates EP-CF and EP-MC2-CF. (**a**) mass; (**b**) mass loss derivative (DTG).

The investigation of the weight fraction of the constituents in the composites was made by means of TGA analysis and the Archimedes’ balance test, which allowed the measurement of the density and porosity. The TGA results are reported in Figure 10a,b and Table S4. The neat epoxy resin (EP) degrades at 368 °C in a single step, also found in all other samples, while the two samples containing MC2 also present the degradation step of docosane, at 210 °C. The residues at the end of the test are mostly due to carbon fibers, but also partly to the shells of the microcapsules and residual char of the epoxy. 

From the comparison of the residues at 700 °C, also reported in Table 4, an experimental fiber weight fraction was determined for the laminates. It can be observed that this value is higher for the EP-CF laminate. This could be attributed to the increase in the matrix viscosity produced by the microcapsule that might prevent the matrix from flowing out of the fabric during the laminate fabrication, thereby favoring a high final matrix weight fraction. With the experimental weight distribution of the composites and the density of each constituent, a theoretical density was calculated and compared with the experimental density obtained via Archimedes’ balance technique. This comparison allowed the calculation of a pore volume fraction, which is comparable for the two laminates and compatible with the adopted hand layup process. Moreover, from the degradation step of docosane, an experimental weight fraction of docosane was determined for the MC2-containing samples. It resulted as 7.6 wt% for the matrix EP-MC2 and 4.3 wt% for the laminate EP-MC2-CF. These values are slightly lower than those determined via DSC, but still in the same range.

#### 3.2.4. Three-Point Bending Test

**Figure 11:**
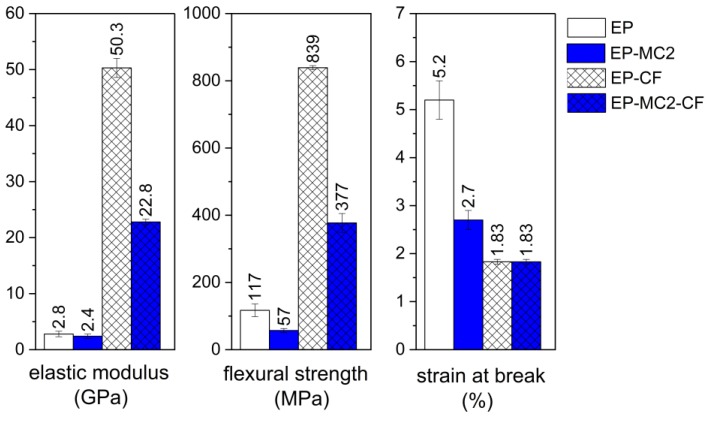
Main results of the three-point bending tests on the epoxy-based matrices EP and EP-MC2, and on the laminates EP-CF and EP-MC2-CF.

The results of the investigation of the mechanical properties are reported in Figure 11. Comparing the samples EP and EP-MC2, the presence of microcapsules decreases the elastic modulus of the epoxy resin only slightly, but it dramatically impairs the properties at break, i.e., the flexural strength and the strain at break. This can be due to the presence of microcapsules aggregates and agglomerates, as observed in the SEM micrographs. A similar trend is also observed in the composites, and although the decrease in mechanical properties are also due to the decrease in the fiber volume fraction, it can be concluded that the introduction of a PCM is not beneficial for the mechanical properties, as was already observed in our previous studies on PCM-enhanced composites [36,37,38,40,41].

#### 3.2.5. DMA Analysis

**Figure 12:**
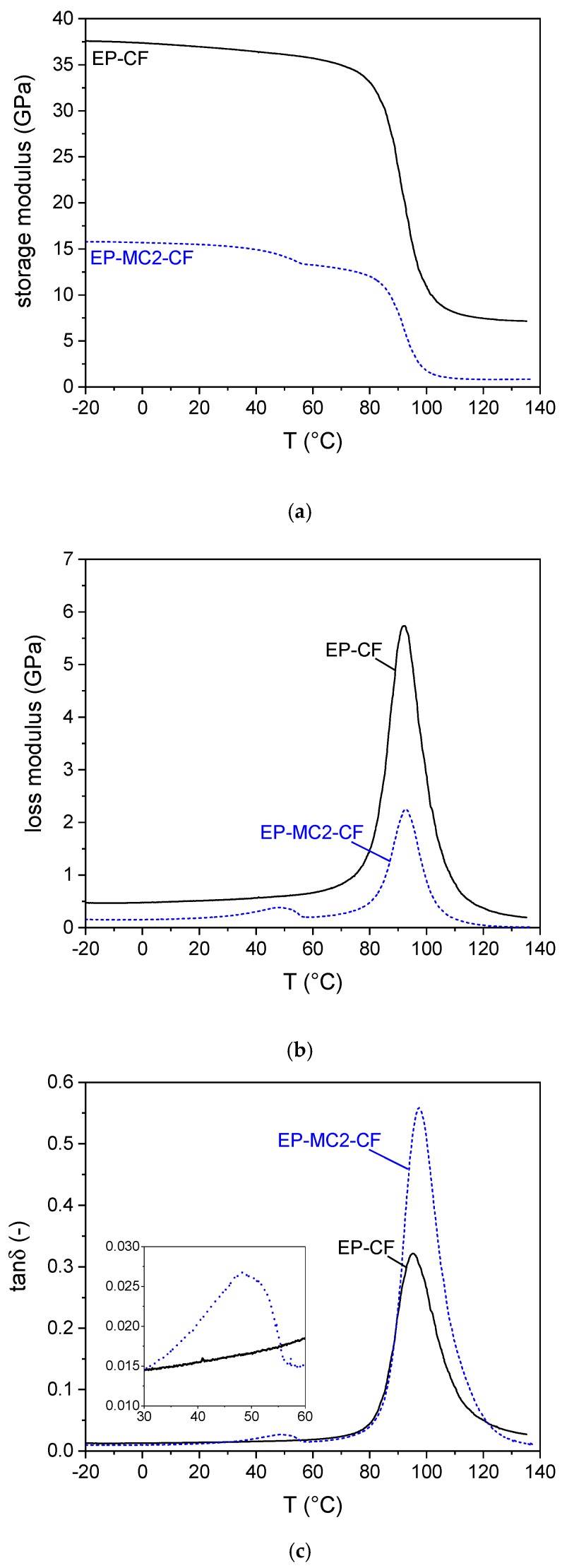
DMA thermograms of the prepared laminates. (**a**) Storage modulus E’, (**b**) loss modulus E”, (**c**) tanδ.

The mechanical and thermo-mechanical properties of the laminates were also assessed through DMA, and the results of the characterization are shown in Figure 12a–c and Table S5. 

Both laminates experience a sharp decrease in the storage modulus in correspondence to the glass transition of the epoxy resin. The position of the glass transition temperature, determined as the peak temperature of the E” or tanδ signals, is not sensibly affected by the presence of the PCM. The laminate containing the microcapsules presents an additional signal at the melting of docosane, evidenced by a step in the storage modulus and non-symmetrical peaks in the trends of E” and tanδ. The storage modulus after the melting phenomenon is approx. 84% of the value at 0 °C, while the sample EP-CF experiences a much smaller decrease over the same temperature interval. These trends in the DMA signals due to a PCM were also observed in previous research of our group [37], and a deeper investigation of these phenomena would be interesting to investigate a melting/crystallization phase change through DMA.

#### 3.2.6. Thermal Camera Imaging

**Figure 13:**
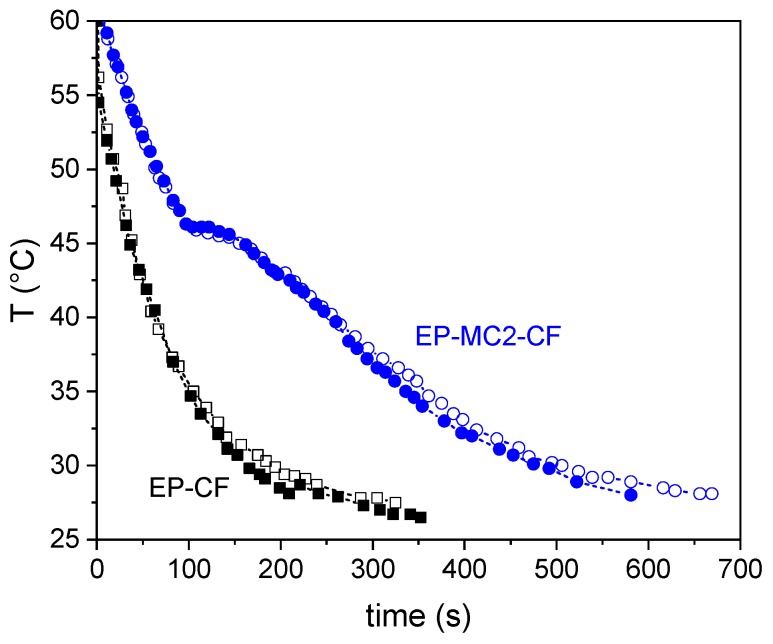
Results of the thermal camera test on the prepared laminates. For reach laminate, the test was repeated on both sides.

A simple test with a thermal camera was performed on the laminates to check their heat storage/management capability. Figure 13 shows the trend of the surface temperature of each laminate upon natural cooling at room conditions. In the sample EP-MC2-CF the temperature decreases with a plateau-like trend induced by the heat released at the crystallization of docosane, which delays the cooling considerably with respect to the sample EP-CF. This test helps in highlighting the potential of PCM-enhanced laminates in thermal management applications.

## 4. Conclusions

In this work, docosane was encapsulated via a sol–gel process in organosilica shells of two different sizes, and the top-performing microcapsules were embedded in an epoxy/carbon laminate to manufacture multifunctional composites combining structural and TES functions. The microcapsules (MC1 and MC2) were extensively characterized and compared to bulk docosane (D) and neat organosilica microspheres (Si). The FTIR spectra of MC1 and MC2 showed all the signals of D and Si, and the analysis suggested that the docosane fraction was considerably higher in MC2 than in MC1, as confirmed by other techniques. The XRD spectrum of MC1 resembled that of the neat organosilica, due to the low docosane fraction, while the spectrum of MC2 contained the patterns of both organosilica and docosane phases, with a probable indication of the presence of the solid rotator phase RII. Solid-state NMR has proved to be a powerful technique to investigate microencapsulated PCMs, providing an in-depth insight into the different microstructures and phases of the samples. In the ^13^C CP-MAS spectra, the sample MC2 showed some minor peaks with an upfield shift of approx. 2 ppm next to the α, β and ε signals, appreciable also in the proton-decoupled spectrum, while the spectrum of MC1 contains only the upfield shifted signals. This shift is related to the presence of a rotator phase or to the reduced docosane chain mobility and interaction with the shell wall (γ-gauche effect). From the areas of α and α’ signals, it was possible to estimate that the rotator phase is the 20% of all the encapsulated alkane for sample MC2, while for the MC1 it represents the 100%. DSC analysis revealed that the melting and crystallization temperatures decreased upon encapsulation, which is due to the difficulty to form perfect crystals in a confined volume and the need for a higher supercooling to start the crystallization. The melting enthalpy measured on MC2, the top-performing microcapsules, was 143 J/g, which implies an encapsulation efficiency of approx. 60%.

In the second part of the work, the microcapsules MC2 were used to produce a structural TES composite. Three-point bending test evidenced a decrease in the mechanical properties of both epoxy and epoxy/carbon laminate after microcapsule addition, associated with the presence of microcapsules agglomerates and the not optimal capsule-epoxy adhesion, both evidenced by scanning electron microscopy (SEM), and dynamic mechanical analysis (DMA) highlighted a decrease of approx. 15% in the storage modulus after docosane melting. The thermal and heat storage properties were investigated through DSC and TGA, and a thermal camera imaging test was useful to assess the thermal management properties and to visualize the heat released during docosane crystallization.

This work contributed to highlight the potentialities of the sol–gel route as a PCM encapsulation technique, to shed light on the change in structural, microstructural and thermal properties due to confinement effect and interaction with the shell wall, and to study the effect of PCM microcapsules on the properties of a carbon/epoxy laminate, in the perspective to produce multifunctional composites. Further studies will be devoted to improving both the emulsion conditions, to increase the encapsulation efficiency, and the microcapsule dispersion in a polymer matrix, to preserve its mechanical properties. Moreover, the thermal reliability of the produced PCM microcapsules will also be evaluated through cyclic DSC tests.

## Figures and Tables

**Table 1:** Composition of the docosane o/w emulsion and the MTES solution for all the prepared samples.

Sample	Docosane o/w Emulsion			MTES Solution		
	Docosane (g)	CTAB (g)	Water (mL)	MTES (mL)	HCl 10^−2^ M (mL)	ethanol (mL)
**MC1**	5	0.05	50	4.2	6.7	5.1
**MC2**						25.5
**Si**	-	-				5.1

**Table 2:** Labeling of the prepared epoxy-based matrices and laminates.

Sample	Composition
EP	Neat epoxy
EP-MC2	Epoxy + MC2 (10 wt%)
EP-CF	Neat epoxy/carbon fiber laminate
EP-MC2-CF	Epoxy/carbon fiber laminate + MC2 (10 wt% with respect to the epoxy)

**Table 3:** Main results of the DSC tests on the bulk (D) and microencapsulated (MC1 and MC2) docosane. The table reports data of the phase change temperatures and enthalpies, as well as the encapsulation efficiency.

Sample	$T_{m}\;{(}^{\circ }C)$	$T_{c}\;{(}^{\circ }C)$	$\Delta H_{m}\;(J/g)$	$\Delta H_{c}\;(J/g)$	η (%)
D	46.2	33.5	234.6	−234.2	100
MC1	41.2	25.1	33.0	−32.9	14.1
MC2	43.8	34.5	142.7	−141.4	60.6

*T_m_*, *T_c_* = melting and crystallization temperatures of the PCM; Δ*H_m_*, Δ*H_c_* = melting and crystallization enthalpies of the PCM; *η* = encapsulation efficiency.

**Table 4:** Density and weight fraction of the constituents of the epoxy-based matrices and laminates from the TGA and Archimedes’ balance tests.

Sample	$R_{700^{\circ }C}$ (wt%)	$\omega_{f}$ (wt%)	$\rho_{exp}$ (g/cm^3^)	$\rho_{th}$ (g/cm^3^)	$\vartheta_{v}$ (vol%)	$R_{260^{\circ }C}$ (wt%)	$\omega_{D}^{TGA}$ (wt%)
EP	6.0	-	1.179 ± 0.001	-	-	97.4	0
EP-MC2	9.4	-	1.089 ± 0.013	-	-	90.1	7.6
EP-CF	66.8	64.7	1.454 ± 0.006	1.508	3.8	98.7	0
EP-MC2-CF	48.2	42.8	1.269 ± 0.008	1.306	2.7	95.9	4.3

$R_{700^{\circ }C}$ = residual mass at the end of the test (700 °C); $\omega_{f}$ = fiber weight fraction; $\rho_{exp}$ = experimental density, measured through Archimedes’ balance method; *ρ_th_* = theoretical density; $\vartheta_{v}$ = pores volume fraction; $R_{260^{\circ }C}$ = residual mass at 260 °C, after the degradation of docosane; $\omega_{D}^{TGA}$ = weight fraction of docosane.

## Supplementary Materials

The following are available online at https://www.mdpi.com/1996-1944/12/8/1286/s1, Figure S1: ^13^C proton-decoupled MAS NMR spectra of docosane (D), MC1 and MC2 microcapsules and the neat organosilica microparticles (Si), Figure S2: T1_C_ curves of the carbons detected in the ^13^C CP-MAS spectra for MC2 (black for original signals, blue spots for interfacial peaks) and MC1 samples (red spots): (**a**)+ (**b**)+ (**c**) chain methylenes from C-3, (**d**) C-2 methylenes, (**e**) methyls, (**f**) MTES methyls, Figure S3: The activation energy for the melting and crystallization processes calculated through the Arrhenius equation (slope of the linear regression). Table S1: T1_C_ relaxation times of the two samples expressed in seconds, Table S2: Main results of the TGA tests on the bulk (D) and microencapsulated (MC1 and MC2) docosane and the neat organosilica microparticles (Si). Table S3: Main results of the DSC tests on the samples EP-MC2 and EP-MC2-CF. The table reports data of the phase change temperatures and enthalpies, as well as the experimental mass fractions of docosane and MC2, Table S4: Main results of the TGA tests on the matrices EP and EP-MC2, and on the laminates EP-CF and EP-MC2-CF, Table S5: Main results of the DMA tests on the laminates EP-CF and EP-MC2-CF.
