# Supplementary material for: Docosane-Organosilica Microcapsules for Structural Composites with Thermal Energy Storage/Release Capability
Source: Materials (Basel). 2019 Apr 19;12(8):1286. doi: 10.3390/ma12081286 (PMC6514850; doi:10.3390/ma12081286)
Supplement: Supplementary file 1 [file materials-12-01286-s001.pdf]

Supplementary materials

Docosane-organosilica microcapsules for structural composites with thermal energy storage/release capability

Giulia Fredi [1,*], Sandra Dirè [1,2,*], Emanuela Callone [1,2], Riccardo Ceccato [1], Francesco Mondadori [1] and Alessandro Pegoretti [1,*]

[1] Department of Industrial Engineering and INSTM research unit, University of Trento, Via Sommarive 9, 38123 Trento, Italy; emanuela.callone@unitn.it (E.C.); riccardo.ceccato@unitn.it (R.C.); francesco.mondadori93@gmail.com (F.M.)

[2] "Klaus Müller" Magnetic Resonance Lab., Department of Industrial Engineering, University of Trento, via Sommarive 9, 38123 Trento, Italy

* Correspondence: giulia.fredi@unitn.it (G.F.); sandra.dire@unitn.it (S.D.); alessandro.pegoretti@unitn.it (A.P.)

Received: 25 March 2019; Accepted: 17 April 2019; Published: date

Supplementary materials

1. Solid state NMR

1.1 Experimental parameters

^{13}C proton-decoupled MAS spectra were acquired under the following conditions: ^{13}C frequency: 100.48 MHz, $\pi/4$ pulse: 2.5 μs, decoupling length: 5.9 μs, recycle delay: 10 s, 300 scans. Samples were packed in 4 mm zirconia rotor and spun at 8 kHz under air flow. Adamantane and Q8M8 were used as external secondary references.

^{13}C spin-lattice relaxation times ($T1_C$) were measured at room temperature with a CP Inversion Recovery Experiment [1] with τ from 1 ms to 30 s and using the already reported CP MAS parameters. The decay curves were fitted with the nonlinear least-square method. The $T1_C$ constant represents the rate needed by the z-component of net magnetization vector (represented by the NMR signal intensity) to go back exponentially to the equilibrium state aligned to the external magnetic field. The process depends on the interaction between the spin system and the close environment. Crystallinity and molecular mobility, together with the closeness to paramagnetic centers or unpaired electrons, strongly affect the relaxation mechanism generally described by the Bloch equation [2].

1.2 Results

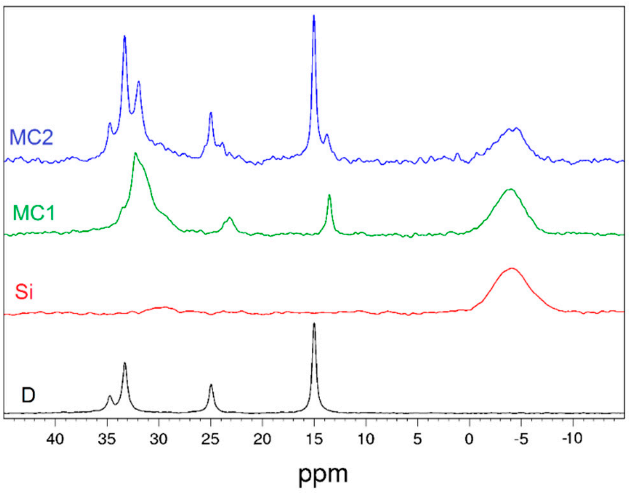

Figure S1. ^{13}C proton-decoupled MAS NMR spectra of docosane (D), MC1 and MC2 microcapsules and the neat organosilica microparticles (Si).

In order to save experimental time, the quantitative analysis can be run considering only the Si-CH_3 and the docosane α-carbons because the recycle time used is not enough to permit the complete relaxation of all the carbon magnetizations. This can be clearly deduced from the relative areas of the C signals in the D spectrum. This is still true for MC2 sample, but no longer for MC1 sample where the CH_2/CH_3 area ratio equal to 9.5 represents correctly the docosane carbon atom distribution. This is a rough indication that the inclusion in small capsules causes a reduction of the T_{1C} of the methylenes of the docosane through interaction with the organosilica shell. Subsequently, in order to quantify this reduction, the relaxation experiments have been done. The mechanism to recover the equilibrium M(t)- M(0) is represented by an exponential decay characteristic of every C in its own functional group. The decay curves of all the carbon signals in MC1 and MC2 spectra are reported in Figure SM2. It is clear that the confinement leads to an acceleration of the process for all the methylene carbons, whereas the methyls from both docosane and MTES are insensitive.

The relative T_{1C} constants are reported in Table SM1.

Table S1. T_{1C} relaxation times of the two samples expressed in sec.

C type	MC1	MC2
γ	61.6	117.0
ε	61.5	109.6
δ	36.5	44.3
β	-	33.7
β′	2.1	4.1
α	-	1.6
α′	1.3	1.2
Si-CH3	5.0	4.6

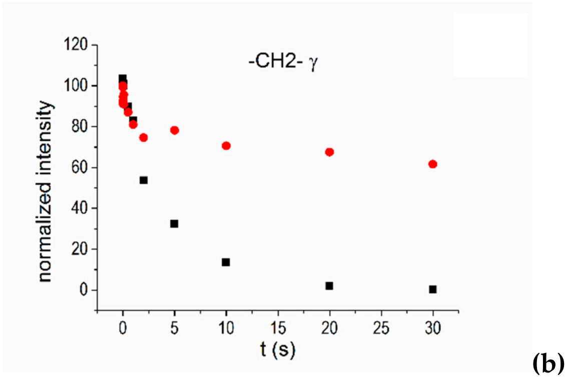

(a)

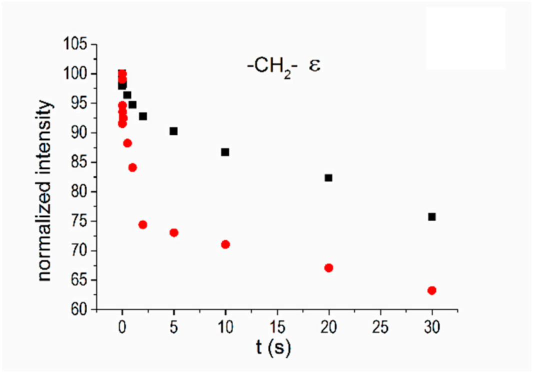

(b)

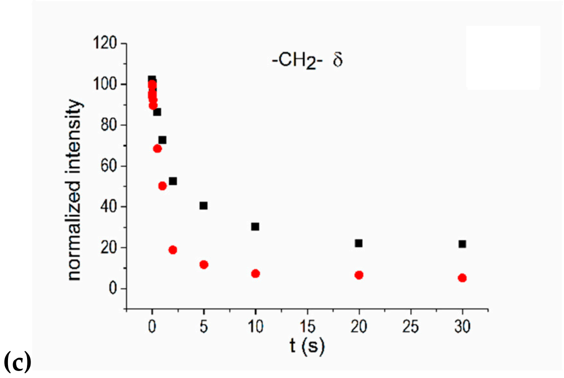

(c)

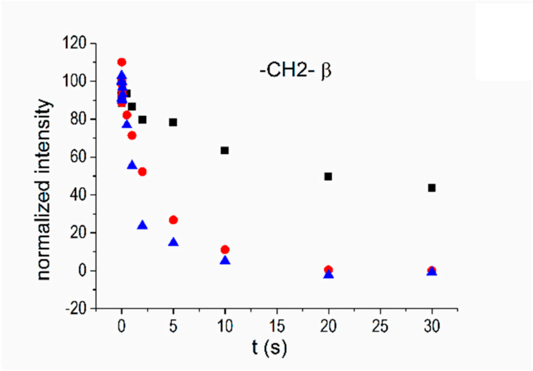

(d)

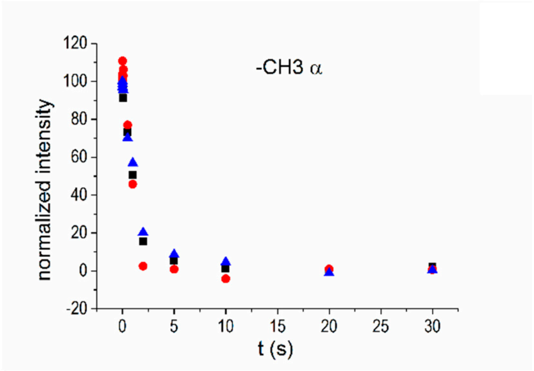

(e)

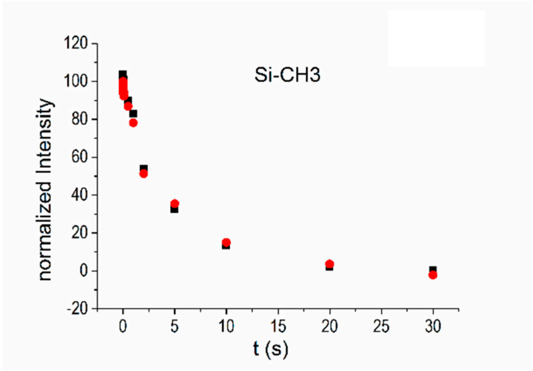

(f)

Figure S2. T1c curves of the carbons detected in the ^{13}C CP MAS spectra for MC2 (black for original signals, blue spots for interfacial peaks) and MC1 samples (red spots): (**a**)+ (**b**)+ (**c**) chain methylenes from C-3, (**d**) C-2 methylenes, (**e**) methyls, (**f**) MTES methyls.

2. DSC and TGA analyses on the microcapsules

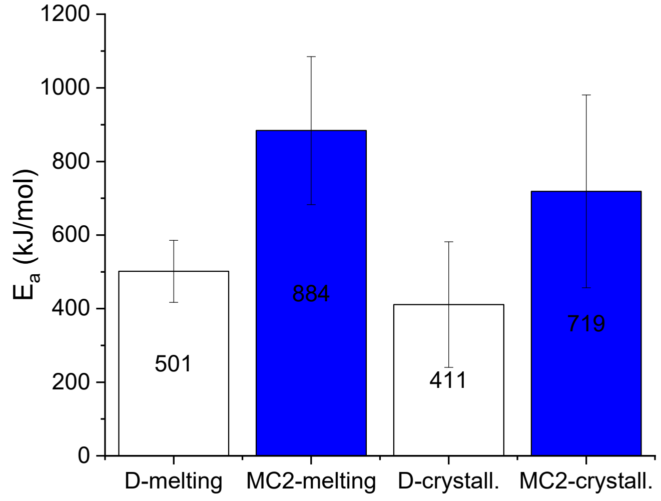

Figure S3. Activation energy for the melting and crystallization processes calculated through the Arrhenius equation (slope of the linear regression).

Table S2. Main results of the TGA tests on the bulk (D) and microencapsulated (MC1 and MC2) docosane and the neat organosilica microparticles (Si).

**Sample**	$T_{1\%}$ **(°C)**	$T_{3\%}$ **(°C)**	$T_{5\%}$ **(°C)**	T_p^D **(°C)**	T_p^{Si} **(°C)**	ω_D^{TGA} **(%)**	$R_{700°C}$ **(%)**
**D**	179.8	203.5	214.8	291	-	100	-
**MC1**	175.3	212.8	227.0	278	572	26	30
**MC2**	156.2	184.0	195.5	255	547	65	10
**Si**	421.8	479.3	498.7	-	563	-	26

$T_{1\%}$, $T_{3\%}$, $T_{5\%}$ = temperatures corresponding to a mass loss of 1 wt%, 3 wt% and 5 wt%; T_p^D= peak temperature of the mass loss derivative, at the degradation of the docosane phase; T_p^{Si} = peak temperature of the mass loss derivative, at the degradation of the organosilica phase; ω_D^{TGA} = weight fraction of docosane; $R_{700°C}$ = residual mass after the test.

3. DSC, TGA and DMA analyses on the epoxy-based matrices and laminates

Table S3. Main results of the DSC tests on the samples EP-MC2 and EP-MC2-CF. The table reports data of the phase change temperatures and enthalpies, as well as the experimental mass fractions of docosane and MC2.

Sample	T_m (°C)	T_c (°C)	ΔH_m (J/g)	ΔH_c (J/g)	ω_D^{DSC} (wt%)	ω_{MC2}^{DSC} (wt%)
EP-MC2	45.3	29.7	14.3	14.6	6.2	10.2
EP-MC2-CF	45.0	35.3	9.3	9.6	4.1	6.7

T_m, T_c = melting and crystallization temperatures of the PCM; ΔH_m, ΔH_c = melting and crystallization temperatures of the PCM; ω_D^{DSC} (wt%) = experimental mass fraction of docosane; ω_{MC2}^{DSC} (wt%) = experimental mass fraction of microcapsules MC2

Table S4. Main results of the TGA tests on the matrices EP and EP-MC2, and on the laminates EP-CF and EP-MC2-CF.

Sample	$T_{1\%}$ (°C)	$T_{3\%}$ (°C)	$T_{5\%}$ (°C)	T_p^D (°C)	T_p^{EP} (°C)
EP	184.7	281.5	327.2	-	368.7
EP-MC2	156.8	192.8	208.5	210.2	378.2
EP-CF	244.3	345.3	363.3	-	385.4
EP-MC2-CF	190.2	226.3	295.5	210.1	387.2

$T_{1\%}$, $T_{3\%}$, $T_{5\%}$ = temperatures corresponding to a mass loss of 1 wt%, 3 wt% and 5 wt%; T_p^D = peak temperature of the mass loss derivative at the degradation of the docosane phase; T_p^{EP} = peak temperature of the mass loss derivative at the degradation of the epoxy phase.

Table S5. Main results of the DMA tests on the laminates EP-CF and EP-MC2-CF.

Sample	$E'_{60°C}/E'_{0°C}$ (%)	$E'_{120°C}/E'_{0°C}$ (%)	$T_{p,E''}$ (°C)	$T_{p,tan\delta}$ (°C)
EP-CF	95.5	19.9	92.2	94.9
EP-MC2-CF	84.3	5.2	92.4	96.8

$E'_{60°C}/E'_{0°C}$ and $E'_{120°C}/E'_{0°C}$ = ratio between E′ values at the indicated temperatures; $T_{p,E''}$ = peak temperature of E″; $T_{p,tan\delta}$ = peak temperature of tanδ.

References

1. Alamo, R. G.; Blanco, J. A.; Carrilero, I.; Fu, R. Measurement of the ^{13}C spin–lattice relaxation time of the non-crystalline regions of semicrystalline polymers by a CP MAS-based method. *Polymer*, **2002**, 43(6), 1857-1865.
2. Bloch, F. Nuclear Induction, *Physical Review* **1946**, 70, 4604–73.
